# Explicit electrothermal LLP VO_2_ model reproducing Preisach like hysteresis for memristive and neuromorphic devices

**DOI:** 10.1038/s41598-026-49919-9

**Published:** 2026-04-24

**Authors:** B. A. S. F. Sena, L. A. L. de Almeida

**Affiliations:** 1 Center for Engineering, Modeling and Applied Social Sciences, Federal University of ABC (UFABC), Santo André, SP 09210-580 Brazil; 2 Department of Electrical, Federal Institute of São Paulo (IFSP), São Paulo, SP 01109-010 Brazil

**Keywords:** Engineering, Materials science, Mathematics and computing, Nanoscience and technology, Physics

## Abstract

Vanadium dioxide (VO$_2$) devices exploit a thermally driven metal–insulator transition accompanied by strong hysteresis, making them promising candidates for compact memristive elements and neuromorphic functionalities. Here we present a fully explicit electrothermal LLP–VO$_2$ model aimed at enabling practical implementation of LLP hysteresis operators in VO$_2$ and related materials. The formulation couples a single thermal balance equation to a discrete LLP operator that governs the metallic fraction while capturing key Preisach-like features, including nested hysteresis loops and return-point memory. The resulting resistance expression combines an Arrhenius-type conduction term with a metallic offset. A systematic comparison between explicit Euler and fourth-order Runge–Kutta (RK4) time integration schemes examines numerical accuracy and solver-dependent stiffness effects across the transition region. The results indicate that LLP-based hysteresis can be simulated efficiently without introducing additional differential states, while maintaining numerical reproducibility. The proposed model may serve as a lightweight and implementation-ready framework for applications requiring controlled VO$_2$ hysteresis behavior, and is designed to be compatible with SPICE-class solvers for future integration and benchmarking against HP- and Chua-type compact models.

## Introduction

Metal–insulator transition (MIT) devices constitute a class of strongly nonlinear electronic components in which electrical behavior emerges from coupled thermal, electrical, and structural processes. These devices are of broad interest across materials science, applied physics, and neuromorphic engineering because their macroscopic response is influenced by microscopic phase transformations. Among known MIT materials, vanadium dioxide (VO$_2$) has long served as a canonical platform due to its sharp semiconductor–to–metal transition and large resistivity contrast^1,2^. These characteristics underpin a wide range of device functionalities, including switching, oscillation, neuristor operation, and memristive behavior, all of which rely critically on the material’s pronounced thermal hysteresis.

Electrothermal origin of switching and hysteresis in VO$_2$. The resistance switching observed in VO$_2$ devices is commonly driven by coupled electrothermal feedback near the metal–insulator transition. Under electrical excitation, the applied current produces Joule heating within the active region, causing the local device temperature to rise. As the temperature approaches the critical transition point $T_c \approx 320-340$ K, VO$_2$ undergoes an insulator–to–metal transformation accompanied by a sharp reduction in resistance. This change in conductivity alters the electrical power dissipation, establishing a nonlinear feedback loop between temperature and resistance. Upon cooling, the reverse metal–to–insulator transition occurs at a different temperature due to intrinsic hysteresis associated with phase coexistence and metastability, leading to distinct heating and cooling branches. Reversals in the thermal trajectory can naturally give rise to nested minor loops and return-point memory, reflecting the multistable character of the transition. Capturing this electrothermal mechanism in a compact and numerically robust form is essential for circuit-level simulation of VO$_2$ switching dynamics and neuromorphic functionalities.

A central challenge in modeling VO$_2$ devices is that their resistance is not a single-valued function of temperature. Polycrystalline films exhibit rich hysteretic behavior comprising major loops, nested and branch-dependent minor loops, and First-Order Reversal Curves (FORC), which reveal multiple metastable states within the transition region^3,4^. This phenomenology is commonly attributed to a distributed ensemble of metallic and semiconducting microdomains whose switching thresholds reflect nucleation barriers, strain, and disorder. Preisach-type formulations naturally capture this structure by representing the material response as a weighted superposition of elementary hysteretic units. Such models were first applied to VO$_2$ by de Almeida et al.^1^ and later refined to account for low-temperature deviations and nonidealities^5^. Despite their descriptive power, direct Preisach implementations scale poorly in time-domain simulations and introduce nonlocal state dependence that complicates coupling to electrothermal solvers.

An alternative and widely adopted modeling strategy relies on compact Chua- and HP-type formulations, in which MIT devices are described through coupled nonlinear ordinary differential equations (ODEs) governing the device temperature and an internal structural state variable associated with the phase transition. These models successfully reproduce pinched *I*–*V* hysteresis loops, local activity, and oscillatory dynamics, and form the basis of the Hewlett–Packard compact model for NbO$_2$ memristors introduced by Pickett et al.^6^. Closely related approaches are extensively used in neuromorphic and nonlinear dynamical demonstrations employing VO$_2$ devices^7,8^.

In ODE-based compact models, however, hysteresis arises implicitly from nonlinear dynamics rather than from an explicit hysteresis operator in the Preisach sense. As a consequence, fundamental hysteretic properties, including return-point memory, branch-dependent minor-loop formation, and accommodation, are not explicitly enforced and can become sensitive to parameter choices and numerical integration details. In addition, the strong stiffness of the coupled thermal–electronic dynamics near the MIT imposes stringent timestep constraints, which can limit large-scale simulations and complicate robust co-simulation within finite-element environments and SPICE-class circuit solvers.

A conceptually distinct alternative is provided by the Limiting Loop Proximity (LLP) operator introduced by de Almeida et al.^9^. LLP offers a closed-form, event-driven approximation to Preisach hysteresis that captures the essential geometrical structure of nested loops and accommodation while remaining algebraic and free of additional differential states. Through an effective-medium formulation, LLP was extended to model the VO$_2$ resistance transition^2^. Because LLP updates are triggered only by reversals of the driving variable, the approach integrates efficiently with time-domain circuit simulators and multiphysics frameworks^10^, while avoiding the stiffness inherent to ODE-based hysteresis descriptions.

Recent work by Zhang et al.^11^ highlighted the potential of hybrid electrothermal models that combine explicit thermal dynamics with an LLP–VO$_2$ hysteresis operator. Euler-based simulations demonstrated long-range ordering and reservoir-computing behavior in large two-dimensional networks. However, the methodology was presented primarily at a conceptual level, with key implementation aspects such as event detection, proximity updates, re-anchoring rules, and numerical safeguards not fully specified, which may hinder reproducibility and broader adoption.

Subsequent studies by Sena and Almeida^12^ employed the LLP–VO$_2$ operator in conjunction with higher-order integration schemes, including Runge–Kutta methods, to simulate coupled electrothermal dynamics under time-varying excitation. In that context, the LLP formalism was applied along continuous temperature trajectories, enabling the reproduction of both major and minor hysteresis loops in transient regimes. Nevertheless, a fully explicit and implementation-ready formulation suitable for direct reuse across simulation platforms remains lacking.

The key contribution of the present manuscript is to provide a fully specified and directly reusable LLP–VO$_2$ implementation, designed for solver portability, including complete event-driven update rules and systematic benchmarking through FORC-based hysteresis validation. Unlike earlier phenomenological LLP-based descriptions of VO$_2$ hysteresis^2^ and our prior transient applications^12^, the present work provides a fully explicit and implementation-ready LLP–VO$_2$ formulation whose hysteresis geometry remains robust across explicit integration schemes, supported by complete event-driven rules and FORC-based validation.

Here we address this gap by presenting a fully explicit and reproducible hybrid electrothermal LLP–VO$_2$ model. The formulation couples a single-state thermal balance equation to an event-driven LLP operator that governs the metallic fraction through a proximity map consistent with Preisach geometry. This structure captures Preisach-like hysteresis features, including accommodation and return-point memory, while avoiding additional stiff internal states or hidden variables. We first perform a systematic comparison between explicit Euler and fourth-order Runge–Kutta (RK4) integration schemes applied to the same thermal system, examining numerical robustness and stiffness effects as the temperature trajectory traverses the steep MIT region. The purpose of this comparison is not to benchmark integration algorithms themselves, but to verify that the geometric structure of the hysteretic response remains invariant under different numerical solvers. Using First-Order Reversal Curve (FORC) analysis, we then illustrate the resulting hysteresis organization and its Preisach-like structure. Beyond VO$_2$, the broader LLP hysteresis framework also provides a principled basis for analyzing relaxation and memory-retention phenomena in thermally driven memristive materials^13,14^.

Overall, this work provides a solver-portable LLP–VO$_2$ electrothermal framework for reproducible circuit-level modeling of hysteretic switching dynamics, offering a practical foundation for future studies of oscillatory, adaptive, and memory-enabled behavior in emerging VO$_2$ hardware platforms. More broadly, it provides a general route for modeling hysteresis in thermally activated memristive materials, enabling reproducible large-scale simulations of phase-transition-based electronic systems.

## Model formulation

The electrothermal behavior of VO$_2$-based devices can be described by the interplay between Joule heating, thermal dissipation, and the hysteretic evolution of the metallic phase fraction associated with the metal–insulator transition. In the present work, we formulate a hybrid electrothermal model in which the device temperature evolves according to a continuous energy-balance equation, while the phase transition dynamics are governed by a discrete hysteresis operator based on the LLP framework. The resulting formulation provides an explicit and numerically tractable representation of hysteretic switching phenomena, including nested loops and return-point memory. The following subsections describe the electrothermal dynamics, the hysteresis operator, and the effective resistance formulation used to couple the phase fraction to the electrical response of the device.

### Electrothermal model

**Figure 1:**
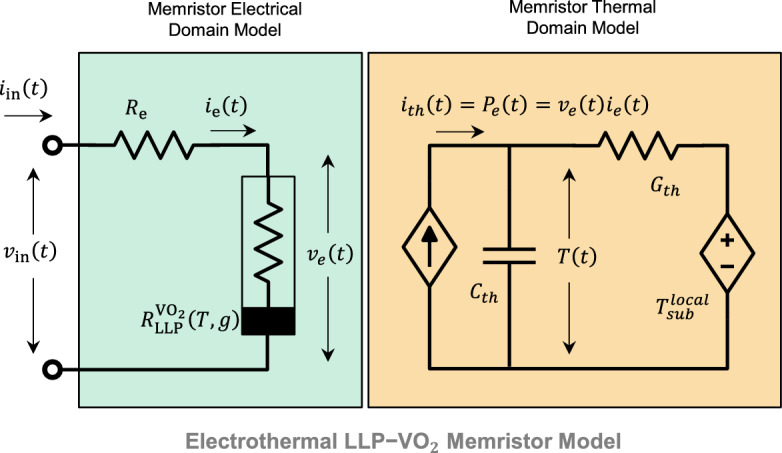
Memristor electrical (left) and thermal (right) domain models, both represented as a memristive device to highlight the coupled electrothermal nature of VO$_2$.

This subsection formalizes the lumped electrothermal balance and the electrical-domain relations employed throughout the simulations. The model couples a single continuous thermal state to an algebraic, history-dependent resistance through the LLP hysteresis operator, yielding a hybrid continuous/discrete structure schematically illustrated in Fig. 1 to clarify the coupled electrothermal structure.

We adopt a single lumped temperature state $T(t)$ representing the film temperature, driven by an externally applied voltage $v_{\textrm{in}}(t)$. The device (electrical-domain) voltage and current are denoted $v_e(t)$ and $i_e(t)$, respectively, and the effective device resistance is written as $R^{\mathrm {VO_2}}_{\textrm{LLP}}\bigl (T(t),g(t)\bigr )$, where $g(t)\in [0,1]$ is the hysteretic metallic fraction.

The instantaneous Joule power delivered to the film is

1 $$\begin{aligned} \begin{aligned} P_e(t)&= v_e(t)\,i_e(t) \\&= \frac{v_e(t)^2}{R^{\mathrm {VO_2}}_{\textrm{LLP}}\,\bigl (T(t),g(t)\bigr )} \\&= i_e(t)^2\,R^{\mathrm {VO_2}}_{\textrm{LLP}}\,\bigl (T(t),g(t)\bigr ) \end{aligned} \quad [\textrm{W}], \end{aligned}$$

and the temperature evolves according to the lumped electrothermal balance:

2 $$\begin{aligned} C_{\textrm{th}}\frac{\textrm{d}T}{\textrm{d}t} = P_e(t) - G_{\textrm{th}}\bigl [T(t)-T_{\textrm{sub}}^{\textrm{local}}\bigr ] \quad [\textrm{W}], \end{aligned}$$

where $C_{\textrm{th}}$ is the thermal capacitance, $G_{\textrm{th}}$ is the thermal conductance to the substrate, and $T_{\textrm{sub}}^{\textrm{local}}$ denotes the local substrate temperature appearing in the thermal-domain model.

The electrical relations (consistent with the left panel of Fig. 1) are

3 $$\begin{aligned} v_e(t)&= v_{\textrm{in}}(t) - R_e\,i_e(t), \end{aligned}$$

4 $$\begin{aligned} i_e(t)&= \dfrac{v_e(t)}{R^{\mathrm {VO_2}}_{\textrm{LLP}}\,\bigl (T(t),g(t)\bigr )}. \end{aligned}$$

Unless otherwise indicated, numerical experiments employ the decaying sinusoidal excitation

5 $$\begin{aligned} v_{\textrm{in}}(t)=6\,\textrm{e}^{-0.4 t}\sin (2\pi t), \end{aligned}$$

which provides a compact stimulus that repeatedly drives the device through the MIT while limiting long-term thermal accumulation. The formulation is not restricted to this specific waveform; alternative inputs yield qualitatively analogous hysteretic responses.

### LLP hysteresis operator

The Limiting–Loop Proximity (LLP) operator used here supplies an explicit algebraic mapping from the temperature history to an instantaneous metallic fraction $g$. Unlike differential hysteresis formulations, the LLP mapping is purely algebraic, direction-aware and event-driven, which facilitates stable coupling to the thermal ODE and efficient implementation in circuit engines.

To define the operator, consider the following parameters and internal variables:

- $T_c$: transition-center temperature determining the midpoint of the phase change,
- *w*: hysteresis width, proportional to the separation between heating and cooling branches,
- $\beta$: steepness parameter regulating the local slope of the transition,
- $\delta \in \{+1,-1\}$: branch orientation ($+1$ for heating, $-1$ for cooling),
- $(T_r,g_r)$: temperature and internal state at the most recent reversal event,
- $T_{pr}$: proximity scale controlling the degree of minor-loop deformation,
- $\gamma$: shape parameter of the proximity kernel.

Minor-loop formation is governed by a smooth proximity kernel that blends the heating and cooling branches while enforcing proper return-to-origin behavior. In the reference implementation the kernel is defined as

6 $$\begin{aligned} P(x)=\tfrac{1}{2}\,\left( 1-\sin (\gamma x)\right) \,\left( 1+\tanh (\pi ^{2}-2\pi x)\right) , \end{aligned}$$

where $x$ denotes a normalized measure of the trajectory’s displacement from the last reversal. For a sampled temperature $T_n$, the normalized displacement and the effective temperature governing the hysteretic response are

7 $$\begin{aligned} x_n = \frac{T_n - T_r}{T_{pr}}, \qquad T_{\textrm{eff},n} = T_n + T_{pr} P(x_n). \end{aligned}$$

The internal state variable $g_n$, representing the instantaneous metallic fraction, is computed algebraically from the shifted temperature:

8 $$\begin{aligned} g_n = \frac{1}{2}+ \frac{1}{2}\tanh \,\Bigl ( \beta \bigl [\delta \tfrac{w}{2} + T_c - T_{\textrm{eff},n}\bigr ] \Bigr ). \end{aligned}$$

Equation (8) yields distinct heating and cooling branches analogous to limiting curves in classical Preisach models, while $T_{pr}$ and the kernel $P(\cdot )$ control controlled minor-loop deformation. The formulation matches the reference routine g_of_T exactly and preserves differentiability with respect to temperature, which is important for stable coupling to thermal ODE solvers.

Overall, the LLP operator delivers a compact, numerically smooth, and computationally efficient representation of electrothermal hysteresis suitable for circuit-level co-simulation.

### Hybrid continuous–discrete implementation

The numerical realization of the LLP–VO$_2$ model follows a hybrid continuous–discrete structure: the thermal dynamics are integrated continuously in time, whereas the hysteresis memory is updated only at discrete reversal events. To ensure causality and solver-invariant behavior, reversal anchors are assigned at the left boundary of each detected event interval, avoiding anticipatory updates that could distort minor-loop geometry. During each macro-step, the hysteresis state variables are frozen and the thermal equation is advanced with a deterministic right-hand side. Intermediate evaluations required by multi-stage schemes (e.g., RK4) are performed through a non-invasive “clone-and-evaluate” mapping, which provides the conductance fraction without modifying the stored hysteresis memory. This architecture prevents unintended state advances during internal solver calls and enables reproducible hysteresis trajectories across explicit and stiff SPICE-class integration schemes.

### Effective resistance formulation

To close the electrothermal system, the hysteretic internal variable $g_n$ is mapped together with the instantaneous temperature $T_n$ onto an effective electrical resistance. This construction follows the standard interpretation of polycrystalline VO$_2$ films as composite media in which semiconducting and metallic microcrystals coexist across the transition region (effective-medium approximation)^2^.

In the insulating regime, electrical transport is thermally activated and is well described by an Arrhenius law,

$$R_s(T)\propto \exp \,\left( \frac{E_a}{k_BT}\right) ,$$

with activation energy $E_a$. For convenience we introduce

$$\Theta _a = \frac{E_a}{k_B}.$$

Under the effective-medium picture, the film resistance can be written as a weighted combination of the insulating and metallic contributions, $R \approx g\,R_s + R_m$^2^. Since $R_s \gg R_m$ in VO$_2$, this reduces to a compact form in which the activated term is modulated by the hysteretic fraction $g_n\in [0,1]$, while the metallic branch provides a finite residual resistance. Accordingly, the resistance law used throughout is

9 $$\begin{aligned} R_n = R(T_n,g_n) = R_s^{\infty }\,g_n\,\exp \,\left( \frac{\Theta _a}{T_n}\right) + R_0. \end{aligned}$$

here $R_s^{\infty }=17~\Omega$ is the semiconducting prefactor obtained from low-temperature $R(T)$ calibration, consistent with the classical VO$_2$ hysteresis model of Almeida *et al.*^2^, and $R_0$ denotes the metallic resistance offset ensuring a bounded resistance in the fully switched state.

For compact notation we define the effective LLP conductance as

10 $$\begin{aligned} G_{\textrm{LLP}}(T) \equiv \frac{1}{R^{\mathrm {VO_2}}_{\textrm{LLP}}\,\bigl (T,g(T)\bigr )}, \qquad \text {so that}\qquad P = V^2\,G_{\textrm{LLP}}(T), \end{aligned}$$

making explicit how the temperature-dependent electrical response enters the thermal power balance.

Together, Eqs. (2), (8), (9), (14) and fully specify the hybrid electrothermal LLP–VO$_2$ model: the temperature $T(t)$ is the sole continuous state, while hysteresis and resistance evolve through algebraic event-driven updates.

The following section presents the numerical results obtained from the proposed electrothermal LLP framework.

## Results

The simulations demonstrate that the proposed electrothermal LLP–VO$_2$ framework reproduces the characteristic hysteretic switching behavior of VO$_2$ devices while preserving the geometric properties expected from Preisach-type systems. In particular, the computed trajectories exhibit return-point memory, stable hysteresis loops, and structured FORC manifolds, indicating that the explicit LLP formulation captures the fundamental structural features of hysteretic phase transitions.

To evaluate the numerical robustness of the approach, the simulations were performed using different explicit time-integration schemes. The analysis focuses on whether the hysteresis geometry and switching dynamics remain consistent under distinct numerical solvers rather than on benchmarking the solvers themselves. In this context, the reference behavior is assessed through the preservation of structural hysteresis properties, while the fourth-order Runge–Kutta (RK4) scheme is used as a practical numerical benchmark for pointwise comparison.

A systematic comparison is therefore conducted between explicit Euler and RK4 integration applied to the same electrothermal system. These simulations allow us to examine how the integration method influences the thermal trajectory, switching dynamics, and numerical stability, particularly in the steep region associated with the metal–insulator transition (MIT).

Additional simulations obtained with stiff and implicit solvers, including Radau and BDF methods, are provided in the Supplementary Information. These results further confirm that the hysteretic response and loop geometry produced by the proposed model remain consistent across different numerical integration strategies.

### Simulation setup

**Figure 2:**
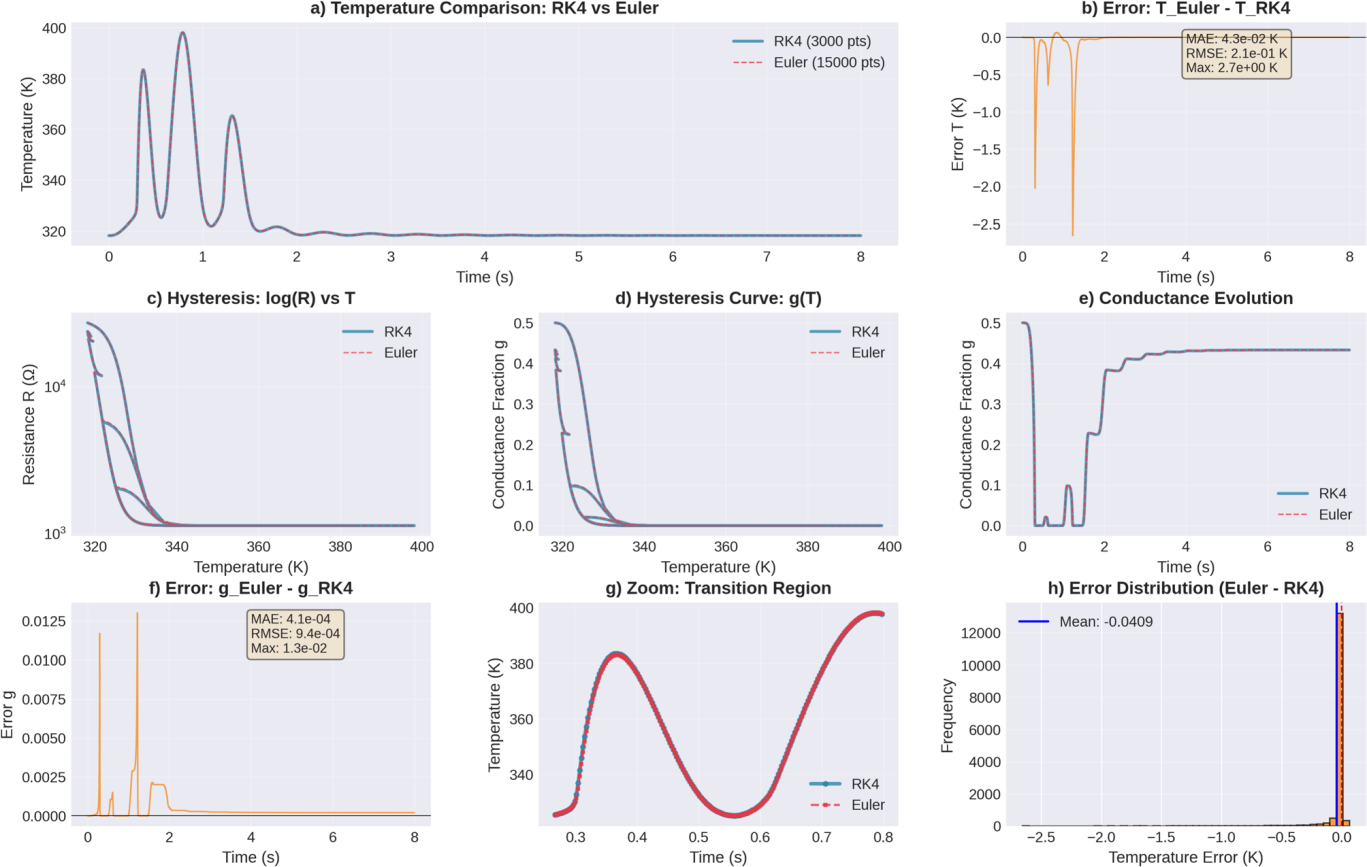
Dynamic comparison between explicit Euler and RK4 integration for the electrothermal LLP–VO$_2$ model under the excitation $v_{in}(t)=6e^{-0.4t}\sin (2\pi t)$, using the parameters listed in Table 1. (**a**) Temperature trajectories. (**b**) Pointwise temperature error, with $\textrm{MAE}=4.3\times 10^{-2}$ K, $\textrm{RMSE}=2.1\times 10^{-1}$ K, and maximum error of $2.7\times 10^{0}$ K. (**c**) Hysteresis loop represented as $\log (R)$ versus *T*. (**d**) Nonlinear conductance function *g*(*T*). (**e**) Temporal evolution of the conductance *g*. (**f**) Numerical error $g_{\textrm{Euler}}-g_{\textrm{RK4}}$, with $\textrm{MAE}=4.1\times 10^{-4}$, $\textrm{RMSE}=9.4\times 10^{-4}$, and maximum error of $1.3\times 10^{-2}$. (**g**) Zoomed view of the phase-transition region. (**h**) Statistical distribution of the temperature error.

**Figure 3:**
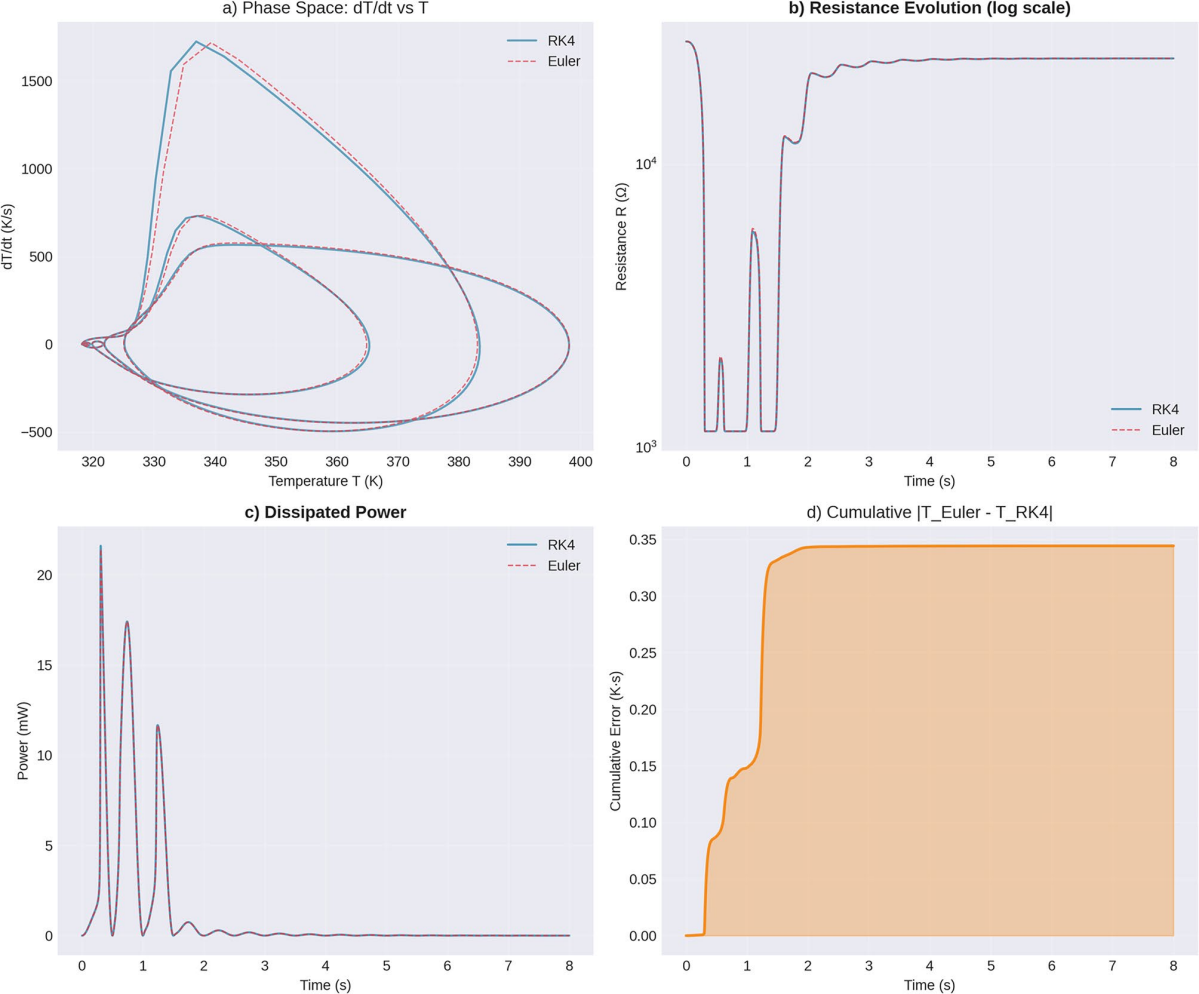
Phase-space and dynamic analysis under explicit Euler and RK4 integration. (**a**) Phase portrait of $\textrm{d}T/\textrm{d}t$ versus temperature. (**b**) Resistance evolution in logarithmic scale. (**c**) Dissipated power. (**d**) Cumulative temperature difference $T_{\textrm{Euler}}-T_{\textrm{RK4}}$.

All results reported in Figs. 1, 2 and 3 were generated using the electrothermal LLP–VO$_2$ model described in the Model formulation section. The simulations couple a lumped thermal balance equation to the event-driven LLP hysteresis operator governing the metallic fraction.

Unless otherwise stated, simulations start from $T(0)=T_{sub}=318.15$ K with $g_0=0.5$ and are driven by the decaying sinusoidal excitation $v_{in}(t)=6e^{-0.4t}\sin (2\pi t)$, which repeatedly drives the system across the metal–insulator transition region. Model parameters are listed in Table 1, while the full numerical implementation details are provided in the Methods section.

The following results compare explicit Euler and fourth-order Runge–Kutta (RK4) integration applied to the same electrothermal system in order to assess whether the hysteresis geometry and switching dynamics remain consistent across different numerical solvers.

### Numerical comparison between Euler and RK4 integration

The simulations show that both explicit Euler and RK4 integration reproduce nearly identical electrothermal switching trajectories and hysteresis loops. Numerical discrepancies remain strongly localized around the metal–insulator transition (MIT), where the electrothermal dynamics become most nonlinear.

Figure 2(a)–(h) summarize the time-resolved comparison between the two integration schemes. Panels (a) and (b) report the temperature trajectories and their pointwise mismatch. The error remains strongly localized in time and is quantified by a maximum deviation of approximately $2.7$ K (see also the statistical metrics reported in the next subsection).

The corresponding hysteretic response is illustrated in panels (c) and (d), which display the resistance–temperature loop in logarithmic scale and the normalized conductance function $g(T)$. Both integration schemes produce nearly indistinguishable hysteresis loops, confirming that the geometric structure of the hysteretic response is preserved across different numerical solvers.

The temporal evolution of the conductance and the solver-to-solver discrepancy are shown in panels (e) and (f). A magnified view of the transition region is provided in panel (g), highlighting the narrow interval where numerical differences become visible. Panel (h) summarizes the statistical distribution of the temperature error obtained across the simulation window.

Figure 3(a)–(d) complement this comparison by reporting the phase portrait, resistance evolution, dissipated power, and the cumulative temperature difference between the two schemes. These representations confirm that both integration methods capture the same global dynamical trajectory and switching sequence.

Importantly, the obtained switching characteristics remain physically consistent with experimentally reported VO$_2$ thin-film devices. The simulated resistance modulation spans several orders of magnitude across the MIT, and the transition occurs around $T_c \approx 320$ K with a hysteresis width of approximately $5$–$10$ K, consistent with experimentally reported ranges in the literature^2,15^.

### Temperature statistics and integration accuracy

Quantitative error metrics further confirm the close agreement between the two integration schemes. Both solvers reproduce the global thermal trajectory and detect identical switching and reversal events, indicating that the underlying hysteresis dynamics remain unchanged.

Numerical discrepancies remain confined to the MIT region, where the strong temperature dependence of the conductance sharply increases the electrothermal feedback and effectively reduces the local thermal time constant. A quantitative comparison yields

$$\textrm{MAE}=4.3\times 10^{-2}\ \textrm{K}, \quad \textrm{RMSE}=2.1\times 10^{-1}\ \textrm{K}, \quad \textrm{max error}=2.7\ \textrm{K}.$$

Outside the transition interval, the thermal dynamics evolve smoothly and both schemes track the relaxation behavior with negligible discrepancy. Within the MIT window, however, the sharp increase in the slope of the LLP conductance function locally amplifies truncation errors, explaining the spatially confined error pattern observed in Fig. 2(b).

Overall, the small numerical differences observed between Euler and RK4 indicate algorithmic reproducibility of the proposed hysteresis operator, rather than a lack of model sensitivity. The explicit LLP formulation therefore preserves the geometric properties of the hysteretic response while remaining robust across different numerical integration schemes.

### Conductance fraction and hysteresis integrity

The conductance fraction dynamics provide a direct visualization of the hysteretic switching process governed by the LLP operator. As illustrated in Fig. 2(c)–(d), the corresponding *R*(*T*) and *g*(*T*) trajectories reconstruct well-defined hysteresis loops across the entire switching cycle.

Nested minor loops and return-point memory are preserved at the algorithmic level, with no detectable drift or distortion of loop geometry. This behavior confirms that the hysteretic dynamics are governed by the internal reversal structure of the operator rather than by the pointwise accuracy of the thermal trajectory.

This robustness originates from the event-driven structure of the LLP operator, in which hysteretic memory is governed by reversal detection rather than by pointwise temperature accuracy. As a result, the switching history remains consistent even when small numerical discrepancies arise in the electrothermal trajectory.

### Phase space, resistance, and power dynamics

The phase-space representation provides additional insight into the nonlinear electrothermal dynamics of the system. As shown in Fig. 3(a), the trajectory in the $(T,\dot{T})$ plane follows a smooth curved path shaped by the coupling between Joule heating and temperature-dependent resistance.

The resistance evolution in Fig. 3(b) exhibits abrupt variations during the metal–insulator transition, which correspond directly to the power transients reported in Fig. 3(c). These rapid variations reflect the strong electrothermal feedback occurring during the phase transition.

Figure 3(d) further indicates that numerical error accumulates predominantly within the same operating region where resistance and power dynamics vary most abruptly. In other words, temperature sensitivity, resistance modulation, power dissipation, and numerical error are all co-localized around the phase-transition regime and originate from the same underlying electrothermal nonlinearity.

The dissipated power is reported to highlight the direct connection between resistance modulation, local Joule heating, and the energetic cost of switching, which is an important consideration for memristive and neuromorphic applications based on VO$_2$ devices.

### Waveform robustness and independence

To further evaluate the robustness of the numerical schemes under different driving conditions, an additional simulation was performed using a damped triangular excitation defined by Eq. (11).

Unlike the damped sinusoidal excitation used throughout the main text and Supplementary Material, which is smooth and continuously differentiable, the triangular waveform introduces abrupt slope changes at each half-period. This creates a more demanding numerical scenario, particularly near the metal–insulator transition where electrothermal feedback amplifies nonlinear effects.

This triangular input is used here only as an additional robustness test, while all other simulations in this work employ the damped sinusoidal excitation described previously.

**Figure 4:**
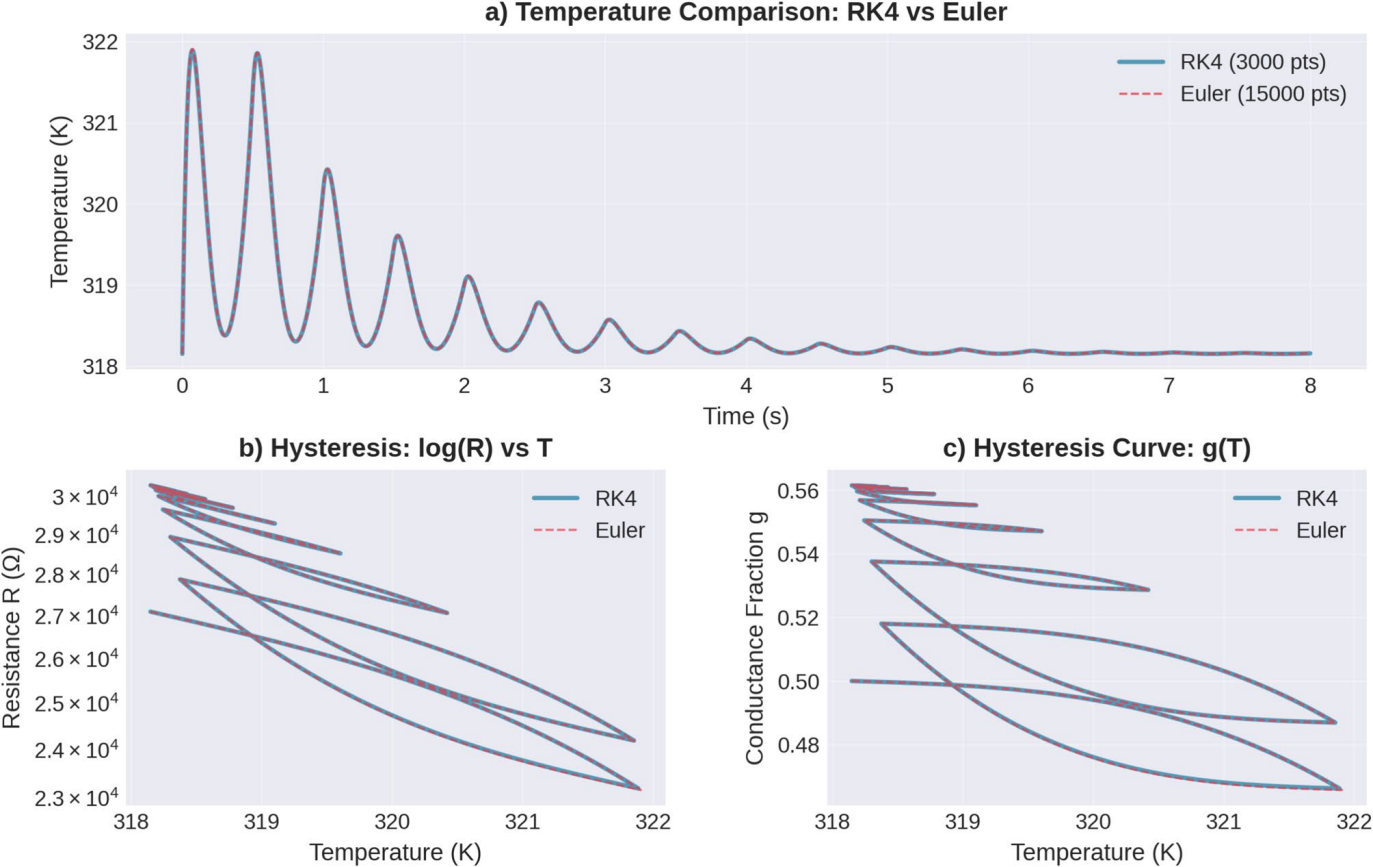
Dynamic comparison between the explicit Euler and RK4 integration schemes under a damped triangular excitation. (**a**) Temperature trajectories. (**b**) Hysteresis loop represented as *R* versus *T*. (**c**) Nonlinear conductance function *g*(*T*).

The results, shown in Fig. 4, demonstrate that the agreement between explicit Euler and RK4 integration remains strong despite the piecewise-linear nature of the excitation. The temperature trajectories closely overlap and the hysteresis loops preserve their structure throughout the transient regime.

These observations indicate that the numerical stability and hysteretic dynamics of the proposed electrothermal framework remain robust under non-smooth waveform excitations, supporting the portability of the model across different driving conditions relevant to experimental VO$_2$ switching devices.

11 $$\begin{aligned} V(t) = 6 e^{-0.4t}\,\textrm{sawtooth}(2\pi t,\text {width}=0.5). \end{aligned}$$

### First-order reversal curve (FORC) response

The first-order reversal curve (FORC) analysis provides an additional structural validation of the Preisach-like hysteresis reproduced by the explicit electrothermal LLP–VO$_2$ model. The resulting family of reversal curves reconstructs a dense hysteresis manifold with well-defined nested minor loops and branch-dependent switching, reflecting the underlying memory structure characteristic of Preisach-type systems.

**Figure 5:**
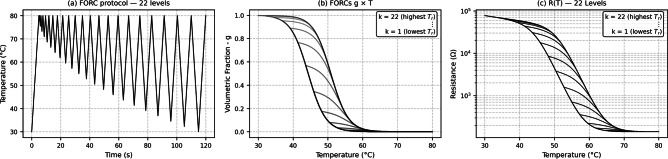
FORC protocol and corresponding hysteretic response. (**a**) Temperature protocol showing repeated cooling–heating cycles with progressively deeper cooling excursions from the maximum temperature. (**b**) Volumetric phase fraction *g*(*T*). (**c**) Resistance *R*(*T*). The first cycle produces the lowest curve, and successive cycles with lower reversal temperatures generate curves that appear progressively higher, establishing a bottom-to-top ordering that follows the sequence of the applied protocol.

Figure 5(a) shows the temperature protocol used to generate the FORC dataset, consisting of 22 uniformly spaced reversal levels spanning the metal–insulator transition (MIT) region. The protocol is implemented as repeated cooling–heating cycles, each starting from the maximum temperature, cooling to a progressively lower reversal temperature, and then reheating back to the maximum temperature.

The first cycle involves only a shallow cooling excursion and therefore produces the lowest curve in Fig. 5(b) and (c). Each subsequent cycle reaches a lower reversal temperature, generating curves that appear progressively higher in the figures. The curves are thus ordered from bottom to top according to the sequence of reversal cycles and the depth of the cooling excursion.

Figure 5(b) and (c) report the extracted volumetric metallic fraction $g(T)$ and the corresponding resistance–temperature characteristics $R(T)$ for each reversal branch. Together these curves form a structured family of nested minor loops that map the accessible hysteretic states of the system.

Importantly, each branch remains uniquely indexed by its reversal temperature, and no drift or degradation is observed across successive cycles. This behavior confirms that the hysteretic response of the LLP–VO$_2$ model is governed by an explicit geometric structure consistent with Preisach-type hysteresis, rather than by emergent solver-dependent dynamics.

### First-order reversal curves (FORCs): experiment and model

To experimentally validate the proposed model, simulations were performed using experimental data obtained from first-order reversal curve (FORC) measurements of the resistance as a function of temperature, *R*(*T*). The experimental dataset was originally acquired by L. A. L. de Almeida in 2000 using instrumentation developed during his PhD at the Laboratório de Instrumentação Eletrônica e Controle (LIEC), Universidade Federal de Campina Grande (UFCG) (https://liec.ufcg.edu.br/). Supporting materials describing the experimental platform in detail are also available in the associated repository.

**Figure 6:**
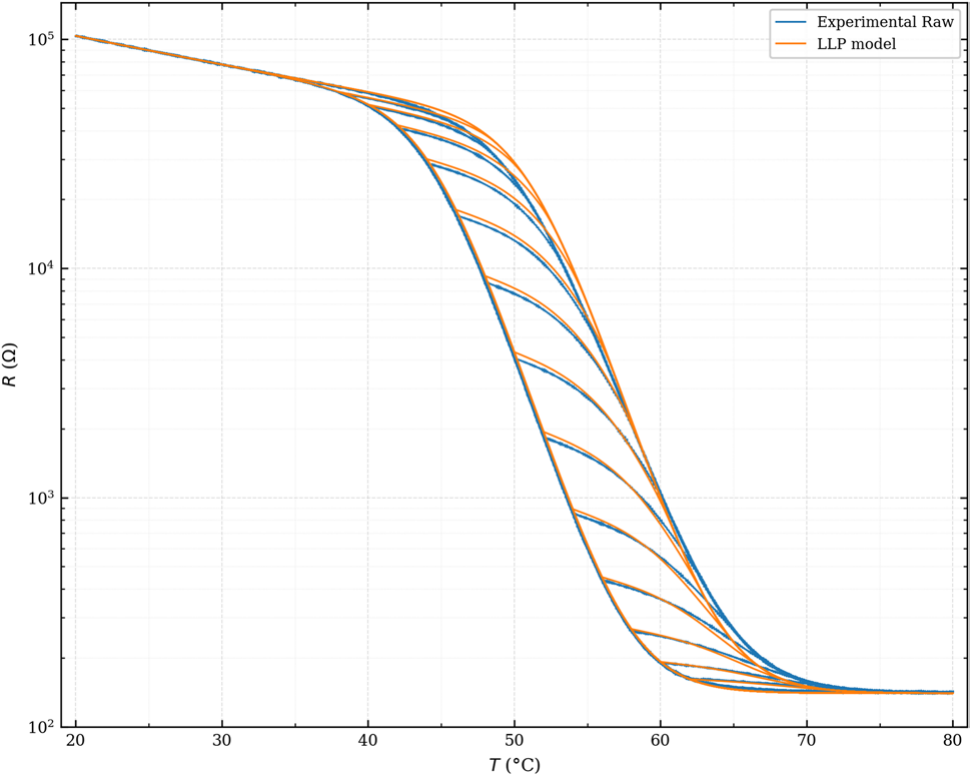
Experimental and modeled first-order reversal curves (FORCs) of resistance as a function of temperature, *R*(*T*). Experimental data (blue markers) are compared with model predictions (orange solid lines). The model captures the progressive shift of the reversal trajectories and their convergence at higher temperatures, reproducing the main hysteretic features of the transition.

Figure 6 presents the experimental FORCs alongside the corresponding model predictions. The experimental data are shown as discrete markers, while the solid lines represent the simulations. Overall, the model accurately captures the progressive displacement of the reversal trajectories as well as the convergence of the curves at higher temperatures, reproducing the key hysteretic features of the phase transition.

In particular, the close agreement between experimental and simulated curves indicates that the proposed formulation successfully describes the evolution of the metallic fraction during partial thermal cycling. Small deviations observed in the intermediate temperature range are consistent with the intrinsic variability of the experimental data and do not compromise the overall fidelity of the model.

These results demonstrate that the model reproduces the main structure of the experimental FORCs without the need for additional internal state variables, reinforcing the physical consistency and predictive capability of the electrothermal LLP–VO$_2$ formulation.

All raw data, scripts, and supporting materials used to generate Figs. 2, 3, 4, 5 and 6 are publicly available at 10.5281/zenodo.19462650

## Discussion

The results presented in this work demonstrate that an explicit electrothermal hysteresis formulation based on the LLP operator can reproduce the key structural features of VO$_2$ switching dynamics while remaining numerically robust and computationally lightweight.

The FORC analysis demonstrates that the explicit LLP–VO$_2$ model captures the characteristic Preisach-like hysteresis structure associated with the metal–insulator transition, including branch dependence, return-point memory, and families of nested minor loops, while remaining computationally efficient. These behaviors are consistent with experimentally reported switching characteristics of VO$_2$ thin films, where distributed transition thresholds give rise to pronounced hysteresis and complex minor-loop structures across the metal–insulator transition.

The family of first-order reversal curves reported in Fig. 5 demonstrates that the hysteretic response is consistent with a well-defined geometric structure, rather than arising from solver-dependent numerical artifacts. This distinction is central to the interpretation of the numerical results: in the present framework, hysteresis is not a byproduct of stiff differential equations, but an explicit and controlled operator embedded within an electrothermal model.

A key implication of this formulation is the clear separation between continuous thermal dynamics and discrete hysteretic memory. The temperature evolves according to a single nonlinear ordinary differential equation, whereas hysteresis enters exclusively through algebraic, event-driven updates tied to reversal points. This separation enables a transparent interpretation in terms of local thermal time scales. Away from the MIT, the conductance varies weakly with temperature and the dominant time constant is set by the ratio $C_{\textrm{th}}/G_{\textrm{th}}$, resulting in slow, non-stiff relaxation. Within the transition region, however, the steep slope of the LLP conductance function locally contracts the effective time scale by orders of magnitude. This contraction explains why numerical discrepancies between Euler and RK4 integration are sharply localized around the MIT, as observed in the Results, and why higher-order schemes achieve superior accuracy without altering the hysteresis topology.

The numerical comparison between Euler and RK4 integration further clarifies accuracy and efficiency trade-offs. RK4 offers higher accuracy per function evaluation and is therefore well suited for single-device studies or operating conditions that traverse the steepest region of the MIT. Explicit Euler, by contrast, remains effective for large-scale simulations when step sizes are chosen conservatively. The strongly localized nature of stiffness suggests that adaptive refinement or stiffly stable implicit schemes may be employed selectively near the transition region rather than uniformly across the trajectory. This observation is particularly relevant for SPICE-class circuit solvers, where backward-differentiation or Radau methods can be introduced without compromising the hysteresis structure imposed by the LLP operator.

Crucially, these solver-induced thermal deviations do not significantly propagate into the hysteretic state. Because reversal detection depends primarily on the direction of the discrete temperature evolution, the LLP operator preserves the sequence of reversal events and the geometry of both major and minor loops across integration schemes. This robustness across integration schemes contrasts with Chua-type and HP-type compact models, in which hysteresis emerges implicitly from stiff coupled ODEs and can become sensitive to discretization or parameter variations. In the LLP–VO$_2$ formulation, hysteresis geometry is enforced at the algorithmic level, ensuring that memory effects remain reproducible across numerical methods and time steps.

From a system-level perspective, these properties are highly relevant for memristive and neuromorphic computing applications. The absence of additional internal differential states reduces stiffness and computational overhead, facilitating the simulation of large arrays and recurrent networks. Predictable hysteresis geometry supports controlled weight programming, plasticity mechanisms, and hardware-aware training strategies, where consistent minor-loop behavior is essential. In addition, the single-state thermal dynamics integrates naturally with neuristor oscillators, relaxation circuits, and time-domain computing primitives, limiting solver-induced distortions when many nonlinear elements are coupled. These properties suggest that the LLP–VO$_2$ formulation can serve as a useful device-level building block for neuromorphic and adaptive circuits, where reproducible hysteresis, controlled nonlinearity, and numerical robustness are essential for large-scale simulation and co-design.

### Limitations and scope

The LLP–VO$_2$ formulation employed in this work is intended to reproduce the macroscopic hysteretic resistance response of VO$_2$ switching devices across the metal–insulator transition within a computationally efficient and implementation-ready electrothermal framework. In this formulation, hysteresis is represented through a reduced-order LLP operator embedded in a lumped thermal balance, providing a purpose-built surrogate for the terminal resistance–temperature (*R*–*T*) behavior at the device level. Although switching in VO$_2$ is known to involve a martensitic structural transition and to be influenced by microscopic mechanisms such as interfacial friction, grain boundaries, and phase coexistence, the present model is not derived from a microphysical free-energy or Landau-type description of the M1$\leftrightarrow$R transition, nor does it aim to explicitly capture nucleation, domain-growth, or spatial percolation dynamics.

Preisach-like hysteresis is nevertheless generally compatible with experimentally reported characteristics of VO$_2$ thin films, where phase coexistence and distributed domain-switching thresholds can lead to multistable transition pathways and nested minor-loop behavior. In this sense, the present framework should be interpreted primarily as an experimentally motivated reduced-order representation of the collective switching dynamics observed in VO$_2$ devices, rather than as a predictive microscopic model of the underlying phase-transition physics.

Although no direct experimental calibration to a specific fabricated device is performed in the present study, the parameter ranges adopted in the LLP–VO$_2$ formulation are chosen to remain consistent with widely reported characteristics of VO$_2$ thin films. In particular, devices typically exhibit a thermally driven transition centered around $T_c \approx 320$–$340\textrm{k}$, accompanied by a resistance modulation spanning several orders of magnitude (340k$10^2$–$10^4$), and a thermal hysteresis width typically in the range of 5–15 K, depending on substrate, film thickness, crystallinity, and microstructural disorder^2,11,15^. The values employed here fall within these reported ranges, supporting the physical plausibility and consistency of the simulated switching behavior and hysteresis organization.            

The LLP-based hysteresis framework was originally introduced and applied to experimental *R*(*T*) characteristics of VO$_2$ by de Almeida et al.^2^, demonstrating that Preisach-like loop organization and minor-loop accommodation provide a compact phenomenological representation of measured switching behavior. More recently, Sena and Almeida^12^ employed the same operator in explicit electrothermal simulations, further supporting its suitability for time-domain circuit modeling under dynamic excitation. In addition, Zhang et al.^11^ incorporated LLP-based modeling to capture thermally driven hysteretic switching in epitaxial VO$_2$ thin films within large-scale neuristor networks. Related hysteretic formulations have also been successfully extended beyond electrical resistance switching, for instance to reproduce optical transmittance modulation across the VO$_2$ transition with good agreement to experimental observations^16^.

The present framework should therefore be interpreted as an experimentally motivated reduced-order model for device-level electrothermal simulation rather than as a quantitative fit to a specific fabricated device. Extensions may be required in regimes where ultrafast dynamics or strongly spatially inhomogeneous transition mechanisms dominate. Future work will focus on systematic parameter extraction from experimental datasets and on extensions incorporating spatially distributed or microstructural effects beyond the present lumped approximation.

Overall, the main contribution of the LLP–VO$_2$ framework lies in combining an explicit hysteresis operator with a reduced electrothermal description, thereby enabling reproducible hysteretic dynamics while maintaining numerical robustness and computational efficiency. By separating hysteretic memory from the continuous electrothermal dynamics, the model clarifies the origin of numerical stiffness near the transition region and localizes numerical sensitivity within the hysteresis operator itself. This separation provides a robust and interpretable foundation for large-scale electrothermal simulations of phase-transition memristive systems, where reproducible hysteresis and computational efficiency are both essential.

## Methods

Given the hybrid electrothermal and event-driven nature of the proposed model, this section provides all necessary details to ensure full reproducibility across numerical platforms.

### Numerical integration methodology

In numerical studies of nonlinear and hysteretic dynamical systems, it is customary to adopt a reference solution against which alternative integration schemes can be evaluated. In this work, the classical fourth-order Runge–Kutta (RK4) method is used as that reference. RK4 is used here as a numerical benchmark for solver comparison, rather than as an experimental ground truth. This choice is not arbitrary, but follows long-established practice in computational physics and numerical analysis^17^, and is particularly well suited to the class of problems addressed here.

The RK4 method provides a favorable balance between accuracy and simplicity. With a global truncation error of order $\mathcal {O}(h^4)$, it achieves a level of accuracy that is already close to the asymptotic solution for sufficiently small time steps, while remaining explicit and straightforward to implement. In contrast to first-order or second-order schemes, such as Euler or midpoint methods, RK4 converges rapidly as the step size is reduced, making it an effective numerical proxy for the “true” solution in problems where no analytical reference exists.

Equally important is the fact that RK4 introduces no solver-side dynamics of its own. Being an explicit, fixed-step method, it does not rely on adaptive time stepping, internal error control, Jacobian estimation, or nonlinear iterations. As a result, the computed trajectories are governed solely by the underlying model equations. This property is essential in systems with hysteresis and event-driven updates, where solver-induced numerical damping or artificial smoothing could otherwise shift reversal points, broaden transitions, or mask physically meaningful fast transients.

The deterministic nature of RK4 further strengthens its role as a reference. For a given time grid and set of parameters, RK4 produces identical trajectories across runs, with a fixed and known number of right-hand-side evaluations per step. This determinism enables clean pointwise comparisons, direct error subtraction, and interpolation onto alternative grids, as implemented in the comparative analysis pipeline of this study (Python code is also available in the associated repository). Such reproducibility is harder to guarantee with adaptive or implicit solvers, whose internal step sequences may vary depending on tolerances and local stiffness.

Although implicit schemes such as BDF or Radau are indispensable when strong stiffness is present, they are not ideal as primary references in this context. Their stability properties, while advantageous for robustness, can suppress or smooth fast modes that are physically relevant in electro-thermal and hysteretic systems. RK4, by contrast, does not presume stiffness and therefore preserves the natural time scales and sharp transitions dictated by the model itself.

Finally, there is a strong element of community convention. Across disciplines ranging from electronic circuit simulation to computational neuroscience and nonlinear dynamics, RK4 is widely accepted as a baseline integrator for benchmarking purposes. Its behavior is well understood, its limitations are transparent, and discrepancies relative to RK4 are straightforward to interpret. For these reasons, RK4 serves as a natural and trusted basis of comparison when assessing the accuracy and numerical behavior of Euler, BDF, Radau, and related integration schemes in scientific reports.

### Event-driven reversal detection

The event-driven logic that updates the LLP memory anchors $(T_r,g_r,T_{pr},\delta )$ is implemented on a discrete time grid. We discretize time as $t_n = n h$ with $T_n \approx T(t_n)$. The branch direction is inferred from the discrete temperature increment

$$\Delta T_n = T_n - T_{n-1},$$

via the rule

12 $$\begin{aligned} \delta _n = {\left\{ \begin{array}{ll} +1, & \Delta T_n > 0,\\ -1, & \Delta T_n < 0,\\ \delta _{n-1}, & \Delta T_n = 0, \end{array}\right. } \end{aligned}$$

which provides a derivative-free indicator of heating or cooling.

A reversal is detected whenever $\delta _n \ne \delta _{n-1}$. At such instances the LLP operator refreshes its memory anchors:

13 $$\begin{aligned} T_r \leftarrow T_{n-1}, \qquad g_r \leftarrow g_{\,n-1}, \qquad \delta \leftarrow \delta _n. \end{aligned}$$

The proximity scale is then updated through

14 $$\begin{aligned} T_{pr} = \delta \frac{w}{2} + T_c -\frac{1}{\beta }\operatorname {arctanh}(2g_r - 1) -T_r, \end{aligned}$$

which computes the gap between the reversal point and the corresponding major-branch temperature and regulates the deformation of the ensuing minor loop. Between reversals the memory variables remain fixed and the hysteresis update is given directly by (8). Because each reversal overwrites the former anchor, the operator enforces the classical wiping-out property and return-point memory while keeping the memory footprint minimal.

### Temperature protocol for FORC simulations

The temperature protocol described below is used to generate the FORC responses reported in Fig. 5.

To obtain the family of first-order reversal curves (FORC), the device is driven by a prescribed temperature program designed to (i) enforce strictly monotonic cooling segments, (ii) ensure a consistent initialization of the VO$_2$ state before each cycle, and (iii) uniformly sample the reversal-temperature space. This construction is designed to ensure that the resulting FORC responses primarily reflect the intrinsic hysteretic dynamics of the model rather than artifacts of the excitation waveform. Because the temperature trajectory is externally prescribed, the resulting FORC responses probe the hysteresis operator under a prescribed temperature trajectory, avoiding solver-dependent electrothermal dynamics.

The protocol starts at the lower temperature $T_{\min }$ and first resets the device by heating it monotonically up to $T_{\max }$, placing the system on the upper (insulating) branch. After this initialization, a sequence of monotonic cooling ramps is applied. Each ramp begins at $T_{\max }$ and is terminated at a prescribed reversal temperature $T_r$, after which the temperature is increased back to $T_{\max }$ to reinitialize the device before the next cycle. This repeated reset–cool–reset structure ensures that each FORC curve is uniquely indexed by its reversal temperature.

Let *n* be the total number of reversal levels. The reversal temperatures are uniformly distributed between $T_{\max }$ and $T_{\min }$ according to

$$\Delta T = \frac{T_{\max }-T_{\min }}{n}, \qquad T_k^{-} = T_{\max } - k\,\Delta T, \quad k=1,\ldots ,n .$$

Each FORC is obtained by cooling the system from $T_{\max }$ down to $T_k^{-}$ at a constant rate $\nu _{\downarrow }$, ensuring $\dot{T}<0$ throughout the descending branch. During this interval, the metallic fraction and resistance are recorded as functions of the instantaneous temperature,

$$g_{\downarrow }(T_r,T), \qquad R_{\downarrow }(T_r,T),$$

with $T_r = T_k^{-}$. Collecting all descending branches for $k=1,\ldots ,n$ yields the complete family of FORC curves parameterized by the reversal temperature.

### Numerical integration schemes

Before presenting the discrete integrators, note that the LLP–VO$_2$ formulation decouples the continuous thermal state from the algebraic hysteresis update. The only dynamical state is the film temperature governed by a first-order ODE; the metallic-fraction update $g(T)$ is algebraic and event-driven. This structure avoids multi-variable stiffness typical of ODE-only compact models and enables the use of standard explicit integrators.

#### Right-hand side

On the discrete grid $t_n = n h$, denote $V_n = V(t_n)$ and define

15 $$\begin{aligned} f(T_n, V_n, g_n) = \frac{1}{C_{\text {th}}} \left[ \frac{V_n^2}{R(T_n,g_n)} - G_{\text {th}}\bigl (T_n - T_{\textrm{sub}}^{\textrm{local}}\bigr ) \right] , \end{aligned}$$

which is the discrete counterpart of the continuous electrothermal ODE. Time-domain simulations are performed over the interval $t\in [0,8]$. The RK4 simulations use a uniform grid of 3000 points, corresponding to a timestep $\Delta t \approx 2.7\times 10^{-3}\,\textrm{s}$. Explicit Euler simulations employ a finer grid of 15000 points ($\Delta t \approx 5.3\times 10^{-4}\,\textrm{s}$) to compensate for the lower-order accuracy of the method and ensure comparable temporal resolution near the metal–insulator transition (MIT).

#### Explicit Euler

The explicit Euler scheme advances temperature as

16 $$\begin{aligned} T_{n+1} = T_n + h\,f(T_n,V_n,g_n), \end{aligned}$$

followed by the LLP hysteresis update described in Sec. LLP hysteresis operator and the event-driven rules of the previous subsection. The fixed step $h$ controls numerical stability and accuracy, particularly near the MIT where the effective thermal time constant is locally reduced.

#### Fourth-order Runge–Kutta (RK4)

We use the classical RK4 procedure with mid-point voltage interpolation:

17 $$\begin{aligned} V_{\text {mid}}^2&= \tfrac{1}{2}(V_n^2 + V_{n+1}^2),\end{aligned}$$

18 $$\begin{aligned} k_1&= f(T_n, V_n, g_n),\end{aligned}$$

19 $$\begin{aligned} k_2&= f(T_n + \tfrac{h}{2}k_1, V_{\text {mid}}, g_n),\end{aligned}$$

20 $$\begin{aligned} k_3&= f(T_n + \tfrac{h}{2}k_2, V_{\text {mid}}, g_n),\end{aligned}$$

21 $$\begin{aligned} k_4&= f(T_n + h k_3, V_{n+1}, g_n), \end{aligned}$$

and

22 $$\begin{aligned} T_{n+1} = T_n + \frac{h}{6}(k_1 + 2k_2 + 2k_3 + k_4). \end{aligned}$$

After the temperature update from either Euler or RK4, the hysteretic state is advanced using the event-driven LLP rules.

### Simulation parameters

The physical, thermal, and hysteresis parameters used in the simulations are summarized in Table 1. The modified parameter set for Fig. 6 is given in Table 2.

**Table 1:** Physical and hysteresis parameters of the LLP–VO$_2$ model.

Parameter	Description	Value
*w*	Hysteresis width	$6.7~\textrm{K}$
$T_c$	Critical temperature	$320.75~\textrm{K}$
$\beta$	Transition slope parameter	$0.25~\mathrm {K^{-1}}$
$\gamma$	Proximity kernel parameter	0.99
$C_{th}$	Thermal capacitance	$1.0\times 10^{-5}~\mathrm {J/K}$
$G_{th}$	Thermal conductance	$2.0\times 10^{-4}~\mathrm {W/K}$
$T_{\textrm{sub}}^{\textrm{local}}$	Substrate temperature	$318.15~\textrm{K}$
$R_0$	Metallic resistance offset	$1140~\Omega$
$E_a$	Activation energy	$0.22~\textrm{eV}$
$k_B$	Boltzmann constant	$8.617\times 10^{-5}~\mathrm {eV/K}$
$g_0$	Initial metallic fraction	0.5

**Table 2:** LLP–VO$_2$ model parameters (only Fig. 6).

Parameter	Description	Value
*w*	Hysteresis width	$6.67~^\circ \textrm{C}$
$T_c$	Critical temperature	$321.05~\textrm{K}$
$\beta$	Transition slope parameter	$0.212~\mathrm {K^{-1}}$
$\gamma$	Proximity kernel parameter	0.9

## Supplementary Information

Supplementary Information.

## Data Availability

Accession codes available at: https://doi.org/10.5281/zenodo.19462650.
