# Supplementary material for: Explicit electrothermal LLP VO2 model reproducing Preisach like hysteresis for memristive and neuromorphic devices
Source: Sci Rep. 2026 Apr 24;16:21682. doi: 10.1038/s41598-026-49919-9 (PMC13358101; doi:10.1038/s41598-026-49919-9)
Supplement: Supplementary file 1 — Supplementary Information. [file 41598_2026_49919_MOESM1_ESM.pdf]

Supplementary Information

for "Explicit electrothermal LLP VO_2 model reproducing Preisach like hysteresis for memristive and neuromorphic devices"

B. A. S. F. Sena and L. A. L. de Almeida

Supplementary Note S1: Physical foundations of the LLP–VO_2 electrothermal framework

The LLP–VO_2 electrothermal framework is intentionally formulated as an effective macroscopic model rather than a microscopic thermodynamic description of the metal–insulator transition. Its physical foundations differ fundamentally from phase-resolved approaches such as the Pickett–Williams model, reflecting both the nature of experimental characterization and the practical requirements of large-scale simulation.

In the LLP approach, thermal conductance G_{th} and thermal capacitance C_{th} are treated as lumped, effective constants. All nonlinearity associated with the metal–insulator transition, including latent heat, domain nucleation, percolation, and phase coexistence, is absorbed into an experimentally measured static resistance–temperature relation $R(T)$ with hysteresis. This relation is obtained under quasi-static thermal control using a thermoelectric module, where the device is driven slowly through heating and cooling cycles until global thermal equilibrium is reached.

Under these experimental conditions, the measured $R(T)$ curve already represents a renormalized response that integrates microscopic thermodynamics, geometric evolution of conductive domains, and microstructural effects inherent to polycrystalline VO_2 thin films. Consequently, the LLP model does not attempt to reconstruct the underlying enthalpy balance or geometric phase evolution from first principles. Instead, it treats the experimentally observed hysteresis as a constitutive property of the device.

Although electrical and thermal conductivities are known to be correlated in VO_2, particularly near the transition, the variation of thermal conductivity is significantly weaker than that of electrical conductivity and is often dominated by heat flow through the substrate and electrode interfaces rather than through the active channel itself. In fabricated devices, the dominant thermal path typically involves the VO_2 film, metal contacts, and substrate stack, whose effective thermal resistance varies little across the transition. Treating G_{th} as constant therefore introduces only a second-order error relative to the dominant electrical nonlinearity captured in $R(T)$.

Similarly, the use of a constant thermal capacitance does not neglect latent heat effects but implicitly incorporates them into an effective thermal inertia. During static characterization, the energy required to traverse the transition is supplied externally by the thermoelectric stage, and the resulting hysteresis width reflects the cumulative enthalpy associated with the phase change. By embedding this information directly into the hysteretic $R(T)$ relation, the LLP framework effectively replaces an explicit enthalpy state variable with an algebraic memory state.

The electrical response of the VO_2 film is modeled using an effective medium approximation (EMA), appropriate for polycrystalline materials with coexisting metallic and insulating domains. Within this framework, the effective conductivity is expressed as a mixture of phase conductivities weighted by an effective metallic fraction, which is inferred from the measured resistance rather than dynamically evolved. This choice aligns more closely with the physical reality of thin-film VO_2 devices than idealized cylindrical or filamentary geometries.

Overall, the LLP–VO_2 model should be interpreted as a reduced-order, experimentally anchored representation of VO_2 hysteresis. It preserves the macroscopic phenomenology of the metal–insulator transition while deliberately sacrificing microscopic phase dynamics in favor of numerical robustness and scalability. This projection of complex thermodynamic behavior onto a hybrid continuous–discrete state space enables efficient electrothermal simulation of large device networks without violating the underlying physics encoded in experimental measurements.

Supplementary Note S2: Numerical Implementation Details

This Supplementary Information provides additional details on the numerical realization of the hybrid electrothermal LLP–VO_2 formulation presented in the main manuscript. In particular, we describe the event-driven hysteresis update strategy and the solver-invariant "clone-and-evaluate" architecture adopted to ensure reproducibility across different classes of integration schemes.

The numerical implementation follows a hybrid continuous–discrete structure in which the thermal dynamics are integrated continuously in time, while the hysteresis memory is updated only at well-defined discrete events. Three design choices are enforced to guarantee causality, reproducibility across integrators, and robustness when the same constitutive block is later transported to stiff SPICE-class solvers.

Hard left anchoring (causal event handling).

Reversal events are detected from the *sign change* of the discrete temperature derivative over successive macro-steps. When a reversal is identified, the hysteresis anchor is established at the *left* boundary of the event interval: the state immediately prior to the direction change, (T_{n-1}, g_{n-1}). This "hard left anchoring" prevents anticipatory behavior (i.e., anchoring at T_n or at an interpolated mid-point), which would otherwise distort minor-loop geometry and bias switching thresholds. In practice, the update rule is: if $\text{sgn}(\dot{T}_n) \neq \text{sgn}(\dot{T}_{n-1})$, then $(T_r, g_r) \leftarrow (T_{n-1}, g_{n-1})$ and the branch direction is updated consistently with the new

sign. The proximity scale is then recomputed from the consistency equation (including the normalization by $P(0)$), ensuring that the internal trajectory remains exactly anchored to the last physically realized state.

Macro-step integration with frozen hysteresis (well-posed ODE evolution).

The thermal equation is advanced over each macro-step $t_{n-1} \to t_n$ while keeping the LLP memory variables fixed. Concretely, the LLP state snapshot

$$\mathcal{S}_k = \{\delta, T_r, T_{pr}, g_r\}$$

is *frozen* at the beginning of the macro-step and is treated as constant throughout the numerical integration used to compute T_n. This decoupling is essential because the electrothermal system naturally combines disparate time scales, and allowing the discrete hysteresis memory to mutate inside a single integration step can introduce solver-dependent artifacts (e.g., multiple unintended re-anchorings during intermediate RK stages or implicit iterations). By freezing $\mathcal{S}_k$, the temperature evolution over $[t_{n-1}, t_n]$ is governed by a deterministic right-hand side, and the hysteresis operator is advanced exactly once per macro-step, at a controlled update point.

Clone-and-evaluate (non-invasive constitutive evaluation).

Within the thermal right-hand side dT/dt, the conductance fraction is evaluated through a *clone* of the hysteresis mapping, computed from the frozen snapshot $\mathcal{S}_k$. That is, whenever the integrator requests an evaluation at an intermediate temperature T (Euler, RK4 substages, or stiff solver internal calls), the code computes

$$g_{\text{clone}}(T; \mathcal{S}_k) = \frac{1}{2} + \frac{1}{2}\tanh\left[\beta\left(\delta_k \frac{w}{2} + T_c - T - T_{pr,k} P\left(\frac{T - T_{r,k}}{T_{pr,k}}\right)\right)\right],$$

and uses it to form $R(T, g_{\text{clone}})$ and hence the Joule heating term. Crucially, this evaluation does **not** modify the true hysteresis memory: $(T_r, g_r, T_{pr}, \delta)$ remain unchanged during the step. This "clone-and-evaluate" architecture makes the simulation *integrator-invariant*: intermediate function calls—whether from RK stages or from the Jacobian/iteration machinery of implicit methods—cannot accidentally advance the discrete memory. As a consequence, the hysteresis update becomes a clean, event-driven layer on top of the continuous thermal evolution, enabling consistent results across explicit and stiff solvers and providing a direct pathway to SPICE-class implementations where the constitutive law must be evaluated repeatedly inside Newton iterations without corrupting state.

Supplementary Note S3: Dimensionality, Computational Cost, and Numerical Stability of VO_2 Memristive Models

Threshold switching in VO_2 devices has been successfully described using physically grounded electro–thermal models, including formulations that explicitly resolve the internal phase evolution within the conductive channel. However, the numerical implications of such detailed descriptions differ substantially from reduced-order hysteresis formulations, such as the LLP–VO_2 model, particularly in terms of dimensionality, stiffness, and scalability.

Two-Dimensional Continuous Dynamics in a Phase-Coexistence Electro–Thermal Model

In phase-coexistence electro–thermal frameworks, the internal state of the device is represented by a continuous variable

$$u(t) = \frac{r_{\text{met}}}{r_{\text{ch}}}, \qquad 0 < u \leq 1, \tag{1}$$

which quantifies the normalized radius of the metallic phase within the channel. This representation captures the radial growth and shrinkage of the metallic region during the insulator–metal transition.

The electrical response of the device is described by a state-dependent Ohmic relation,

$$v(t) = R_{\text{ch}}(u(t))\ i(t), \tag{2}$$

where $R_{\text{ch}}(u)$ is obtained from an effective-medium approximation accounting for radial phase coexistence.

The temporal evolution of u is governed by an electro–thermal power balance,

$$i^2 R_{\text{ch}}(u) = G_{\text{th}}(u)\,\Delta T + \frac{d\Delta H}{dt}, \tag{3}$$

where $G_{\text{th}}(u)$ is the effective thermal conductance and ΔH is the enthalpy associated with the phase transition. Expressing the enthalpy rate as

$$\frac{d\Delta H}{dt} = \frac{d\Delta H}{du}\frac{du}{dt}, \tag{4}$$

leads to the state equation

$$\frac{du}{dt} = \left(\frac{d\Delta H}{du}\right)^{-1}\left[i^2 R_{\text{ch}}(u) - G_{\text{th}}(u)\Delta T\right]. \tag{5}$$

When the device is embedded in a driven electrical circuit containing reactive elements, at least one additional electrical state variable must be introduced. For instance, in a relaxation oscillator configuration, Kirchhoff's current law at the capacitive node yields

$$C\frac{dv}{dt} = \frac{V_{\text{DC}} - v}{R_L} - i(v, u), \tag{6}$$

where the device current i is determined by the state-dependent relation in Eq. (2). The complete model thus forms a coupled two-dimensional system of ordinary differential equations in (v, u).

This continuous 2D formulation must be integrated at every time step, even when the system operates far from the metal–insulator transition. Near the switching threshold, strong positive electro–thermal feedback emerges: an increase in u reduces R_{ch}, which increases the current and Joule heating, further accelerating the growth of u. This feedback results in steep vector fields and widely separated time scales, making the system prone to numerical stiffness and instability under explicit integration schemes.

One-Dimensional Event-Driven Dynamics in the LLP–VO_2 Model

The LLP–VO_2 formulation adopts a reduced-order description in which the hysteretic phase evolution is not treated as a continuously integrated state variable. Instead, the device temperature $T(t)$ is the sole dynamic state governed by

$$C_{\mathrm{th}} \frac{dT}{dt} = P_{\mathrm{Joule}}(T, v, i) - G_{\mathrm{th}}(T - T_{\mathrm{amb}}), \tag{7}$$

while the metallic phase fraction is represented by an algebraic proximity function

$$g(T; \mathcal{S}), \tag{8}$$

where $\mathcal{S}$ denotes a discrete hysteresis state encoding the last reversal temperature and direction.

The electrical resistance of the VO_2 element is then obtained in closed form as

$$R(T) = \mathcal{R}(g(T; \mathcal{S}))\,, \tag{9}$$

and the hysteresis state $\mathcal{S}$ is updated only when a switching or reversal event is detected. Between such events, $\mathcal{S}$ remains frozen, and the resistance law is smooth and single-valued.

As a result, the LLP–VO_2 model reduces the continuous dynamics to a single ordinary differential equation, complemented by discrete event handling. The hysteresis no longer introduces additional stiff modes, as it is not governed by a differential equation but by an event-driven state update.

Computational Cost and Scaling

For a simulation of duration T_{sim} with time step Δt, the computational cost of the continuous electro–thermal phase-coexistence formulation for an array of N devices scales approximately as

$$\mathcal{O}\left(N \frac{T_{\mathrm{sim}}}{\Delta t} c_{\mathrm{2D}} \right), \tag{10}$$

where c_{2D} accounts for the evaluation of nonlinear resistance, thermal conductance, and enthalpy derivatives at each step.

In contrast, the LLP–VO_2 approach yields

$$\mathcal{O}\left(N \left[\frac{T_{\mathrm{sim}}}{\Delta t} c_{\mathrm{1D}} + E\, T_{\mathrm{sim}}\, c_{\mathrm{evt}} \right] \right), \tag{11}$$

where E is the average number of hysteresis events per device per unit simulated time.

Numerical Stiffness and Stability

In continuous electro–thermal phase-coexistence models, stiffness arises intrinsically from the coexistence of fast electrical dynamics and slower, strongly nonlinear thermal and phase evolution. This multiscale structure often necessitates adaptive step-size control or implicit solvers to ensure numerical stability, particularly near the threshold region.

In the LLP–VO_2 formulation, stiffness is primarily associated with the thermal subsystem and circuit parasitics, rather than with the hysteresis itself. By removing the phase evolution from continuous integration and handling it through discrete events, the model avoids numerical chattering and excessive time-step refinement near transition points. Provided that event detection is implemented with appropriate root-finding and state-freezing strategies, the LLP–VO_2 model exhibits improved numerical robustness and scalability.

Supplementary Note S4: Numerical Integration and Solver Stability

Continuous-time integration of both models was performed using standard explicit or adaptive Runge–Kutta methods in non-stiff regimes. In continuous electro–thermal phase-evolution models, numerical stiffness can arise near the metal–insulator transition, and implicit solvers or severely restricted time steps are often required to maintain stability. This behavior is fundamentally linked to the presence of additional internal state variables associated with phase evolution and energy storage, such that the device resistance is not a single-valued function of temperature and hysteresis cannot be reproduced within a purely one-dimensional thermal description without additional memory variables.

In contrast, the LLP–VO_2 framework may be interpreted as a numerically regularized projection of the hysteretic transition onto a hybrid continuous–discrete state space: the thermal balance evolves continuously, while the hysteresis operator updates only at discrete reversal events. As a result, explicit solvers remain stable over a wider range of parameters, enabling efficient large-scale simulation of VO_2 networks while preserving the essential physics of threshold switching and minor-loop organization.

Supplementary Note S5: Dynamic comparison between BDF and RK4 integration schemes.

To further assess solver-dependent numerical effects, we provide an additional dynamic comparison between an implicit backward differentiation formula (BDF) integration scheme and the classical fourth-order Runge–Kutta (RK4) method. Here, RK4 is used as a high-order explicit reference, not as experimental ground truth. Both solvers are applied to the same electrothermal trajectory across the MIT region, allowing a direct evaluation of temperature deviations and their impact on the hysteresis-dependent conductance state.

As shown in Fig. 1(a), both solvers produce closely matching temperature trajectories, with deviations becoming noticeable only during the fast transient near the switching region.

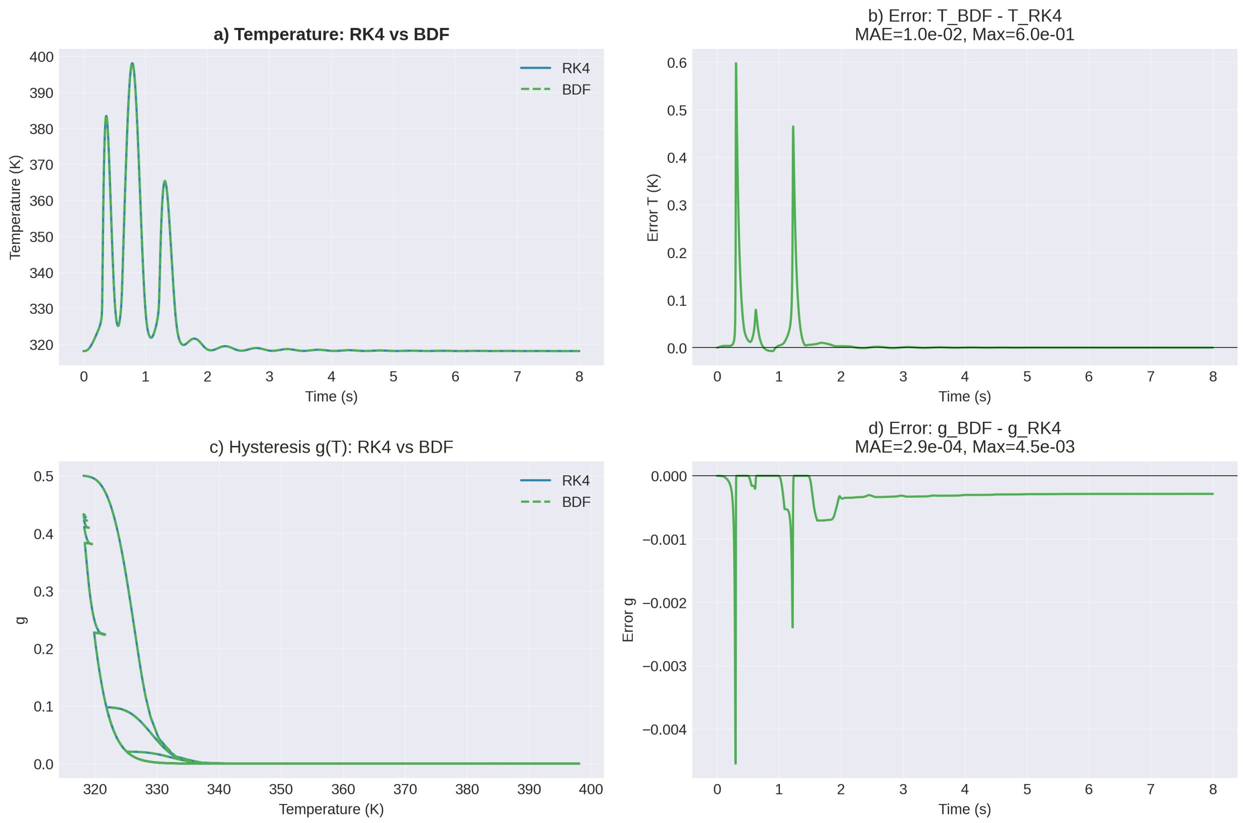

Figure 1: Dynamic comparison between the BDF and RK4 integration schemes. (a) Temperature trajectories. (b) Pointwise temperature error, with MAE $= 1.0 \times 10^{-2}$ K and maximum error of 6.0×10^{-1} K. (c) Nonlinear conductance function $g(T)$. (d) Numerical error $g_{\mathrm{BDF}} - g_{\mathrm{RK4}}$, with MAE $= 2.9 \times 10^{-4}$ and maximum error of 4.5×10^{-3}.

The corresponding pointwise temperature error is reported in Fig. 1(b), where a mean absolute error of MAE $= 1.0 \times 10^{-2}$ K and a maximum deviation of 6.0×10^{-1} K are observed. Despite these local discrepancies, the nonlinear conductance function $g(T)$ remains nearly indistinguishable over most of the trajectory, as illustrated in Fig. 1(c). The residual difference $g_{\mathrm{BDF}} - g_{\mathrm{RK4}}$, shown in Fig. 1(d), confirms that the hysteresis response is only weakly affected, with MAE $= 2.9 \times 10^{-4}$ and a maximum error of 4.5×10^{-3}.

Notably, although temperature deviations are locally amplified during the transient regime, their impact on the hysteresis state remains strongly attenuated due to the nonlinear mapping $g(T)$. These results confirm that, while small discrepancies arise locally, the LLP hysteresis organization remains stable and effectively solver-invariant at the operator level.

Supplementary Note S6: Dynamic comparison between Radau and RK4 integration schemes.

As an additional verification of numerical robustness under stiff integration, we report a dynamic comparison between an implicit Radau integration scheme and the classical fourth-order Runge–Kutta (RK4) method. As in Supplementary Note S5, RK4 is employed purely as a high-order explicit reference rather than as experimental ground truth. The Radau solver is particularly suitable for stiff nonlinear regimes, making it a relevant reference for validating integration stability across the sharp VO_2 transition.

As shown in Fig. 2(a), both solvers produce nearly identical temperature trajectories over the entire simulation window. The corresponding pointwise temperature error, reported in Fig. 2(b), remains extremely small, with a mean absolute error of MAE $= 1.1 \times 10^{-4}$ K and a maximum deviation of 1.4×10^{-2} K. The nonlinear conductance function $g(T)$, shown in Fig. 2(c), is virtually indistinguishable between the two integration schemes. This is further confirmed by the residual difference $g_{\text{Radau}} - g_{\text{RK4}}$ in Fig. 2(d), which remains negligible, with MAE $= 5.3 \times 10^{-8}$ and a maximum error of 4.7×10^{-5}.

These results demonstrate that, even under stiff integration conditions, the LLP hysteresis operator preserves its minor-loop organization and remains effectively solver-invariant. The excellent agreement between implicit and explicit schemes further confirms the numerical robustness and portability of the proposed framework across different classes of time-integration methods.

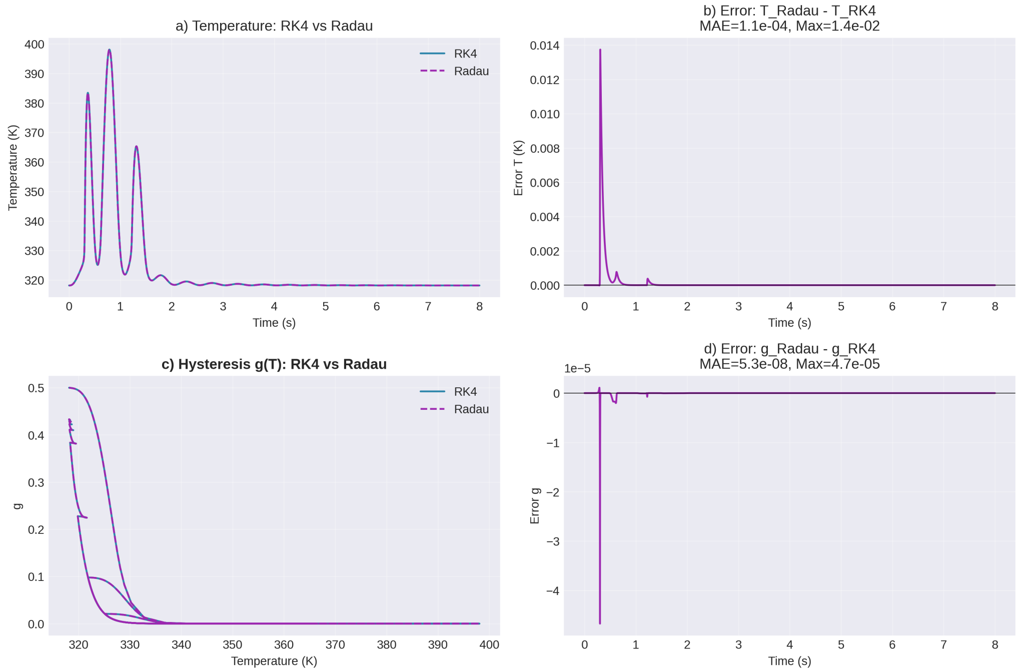

Figure 2: Dynamic comparison between the Radau and RK4 integration schemes. (a) Temperature trajectories. (b) Pointwise temperature error, with MAE $= 1.1 \times 10^{-4}$ K and maximum error of 1.4×10^{-2} K. (c) Nonlinear conductance function $g(T)$. (d) Numerical error $g_{\text{Radau}} - g_{\text{RK4}}$, with MAE $= 5.3 \times 10^{-8}$ and maximum error of 4.7×10^{-5}.
